# Scaling up community mobilisation through women's groups for maternal and neonatal health: experiences from rural Bangladesh

**DOI:** 10.1186/1471-2393-12-5

**Published:** 2012-01-24

**Authors:** Tasmin Nahar, Kishwar Azad, Bedowra Haq Aumon, Layla Younes, Sanjit Shaha, Abdul Kuddus, Audrey Prost, Tanja AJ Houweling, Anthony Costello, Edward Fottrell

**Affiliations:** 1 Perinatal Care Project, Diabetic Association of Bangladesh (BADAS), BIRDEM 122 Kazi Nazrul Islam Avenue Shahbagh, Dhaka-1000, Bangladesh; 2 UCL Centre for International Health and Development, Institute of Child Health, University College London, 30 Guilford Street, London, WC1N 1EH, UK; 3 Department of Public Health, ErasmusMC University Medical Center Rotterdam, Dr. Molewaterplein 50, 3015 GE Rotterdam, The Netherlands

## Abstract

**Background:**

Program coverage is likely to be an important determinant of the effectiveness of community interventions to reduce neonatal mortality. Rigorous examination and documentation of methods to scale-up interventions and measure coverage are scarce, however. To address this knowledge gap, this paper describes the process and measurement of scaling-up coverage of a community mobilisation intervention for maternal, child and neonatal health in rural Bangladesh and critiques this real-life experience in relation to available literature on scaling-up.

**Methods:**

Scale-up activities took place in nine unions in rural Bangladesh. Recruitment and training of those who deliver the intervention, communication and engagement with the community and other stakeholders and active dissemination of intervention activities are described. Process evaluation and population survey data are presented and used to measure coverage and the success of scale-up.

**Results:**

The intervention was scaled-up from 162 women's groups to 810, representing a five-fold increase in population coverage. The proportion of women of reproductive age and pregnant women who were engaged in the intervention increased from 9% and 3%, respectively, to 23% and 29%.

**Conclusions:**

Examination and documentation of how scaling-up was successfully initiated, led, managed and monitored in rural Bangladesh provide a deeper knowledge base and valuable lessons.

Strong operational capabilities and institutional knowledge of the implementing organisation were critical to the success of scale-up. It was possible to increase community engagement with the intervention without financial incentives and without an increase in managerial staff. Monitoring and feedback systems that allow for periodic programme corrections and continued innovation are central to successful scale-up and require programmatic and operational flexibility.

## Background

In line with Millennium Development Goal (MDG) 4, many countries are on track to reduce under-five mortality by two thirds from 1990 levels by 2015 [1]. This progress has not been uniform for all under-five age groups, however. Neonatal mortality has been relatively resistant to change and the 3.7 million neonatal deaths that occur annually worldwide account for an increasing proportion of all under-five deaths [2-5]. In Bangladesh, around 85% of births occur at home, 57% of all under-five deaths are in the first month of life and the neonatal mortality rate remains high at 37 per 1000 live births [6].

Tackling the burden of neonatal deaths, particularly deaths in the first twenty-four hours of life, requires community-based interventions to improve the supply and demand for maternal and neonatal health care and the use of safe home-delivery and newborn care practices that can prevent neonatal deaths [7-9]. Several studies provide encouraging evidence on how home visits or community mobilisation with concurrent health services strengthening can improve maternal and neonatal health in South Asia [10,11]. Sustaining and scaling-up interventions to increase coverage remain critical challenges.

Low-cost, participatory, community-based approaches involving women's groups are effective at improving home delivery practices and birth outcomes in a range of settings. The women's group method significantly reduced neonatal mortality by 30% and 45% in Nepal and India, respectively, and improved hygienic home delivery practices and newborn care in Bangladesh, though it did not have an impact on neonatal mortality overall [7,12,13]. Intervention coverage was one women's group per 756 population and one per 468 population in Nepal and India, respectively [7]. The percentage of women who gave birth and reported attending women's groups was around 30% to 45% in India and 50% in Nepal [12,13]. Coverage in Bangladesh was much lower at one women's group per 1414 population and the proportion of women who delivered and reported attending women's groups was 3%, with just 9% of women of reproductive age becoming women's group members [7].

Program coverage has been observed to be an independent determinant of neonatal mortality, even when adjusted for type of intervention and baseline mortality levels [14]. For community-based interventions to have a substantial impact on birth outcomes, therefore, it is necessary to have a large enough population coverage over a sustained period [14-16]. We hypothesise that the women's group intervention in Bangladesh did not show an impact on neonatal mortality because of its relatively low coverage relative to that used in India and Nepal. For this reason, we increased the coverage in the same geographical areas and the percentage of women in reproductive age and pregnant women exposed to the intervention. The impact of this scaled-up delivery of the intervention is the subject of an ongoing cluster randomised controlled trial detailed elsewhere [17].

Scaling-up is frequently discussed but seldom analysed or rigorously studied [18] yet examination and documentation of how scaling-up experiences are initiated, led, managed and monitored provide a deeper knowledge base and valuable lessons [19]. This paper details experiences in our expansion of women's groups in Bangladesh. We emphasise the importance of monitoring and evaluating the success of scale-up in relation to specific targets, with practical examples of how this may be done in resource-poor settings.

## Methods

### Setting and context

Following from a previous cluster randomised trial [7], scale-up activities were based in nine previously selected unions (the lowest-level administrative level in rural Bangladesh). These unions are located within three districts of Bangladesh (Bogra, Molavibazar and Faridpur) which were selected on the basis of having active Diabetic Association of Bangladesh (BADAS) offices. The majority of the population in the selected areas is Muslim (> 80%), with most of the remainder being Hindu [7]. Most women deliver at home (> 90%) and approximately 50% of mothers in the selected areas have no formal education or only primary education [7]. Physical geography in the three intervention districts, including vulnerability to flooding, contributes to poor communications and limited access to good quality health care.

### Intervention

**Figure 1:**
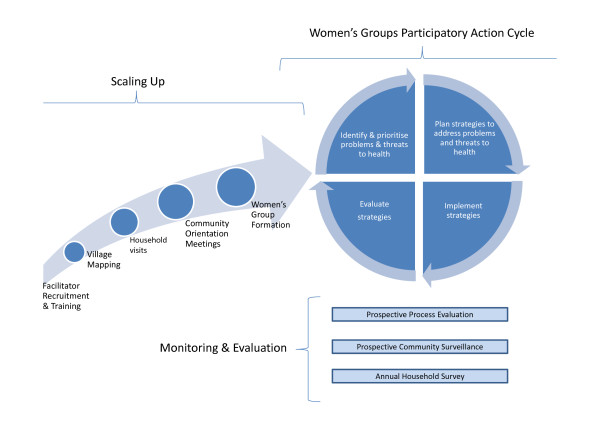
**Schematic illustration of the scale-up process, the participatory action cycle intervention and components of monitoring and evaluating the success of scale-up**.

The intervention is a cycle of monthly women's group meetings on maternal and newborn health and, subsequently, on under-5 and women's health. A salaried facilitator guides the women's groups through a participatory learning and action cycle [20], in which she leads and supports the groups to identify and prioritise their health problems, plan strategies to address them, and implement and evaluate these strategies (Figure 1). All meetings are facilitated with the use of picture cards and flip charts that convey simple health messages and preventative strategies. This intervention content was derived from successful trials in Nepal [12] and India [13] as well as key health issues in Bangladesh. Details of the content and delivery of the intervention are provided elsewhere [21]. Community meetings encourage the participation and support of the wider community in the development and implementation of strategies. Day-to-day implementation of the intervention is decentralised from Dhaka headquarters to district offices. The approximate direct costs of each meeting (i.e. the facilitator, travel and meeting materials) is between 500 and 1000 Bangladeshi Taka (approximately USD 6 to 13 on 16^th ^December 2011).

### Recruitment and training

Figure 1 illustrates the scale-up process. With nine facilitators already active in the intervention areas, a further 36 facilitators were recruited in 2008 to increase population coverage and intensity in the same areas. In recognition of cultural norms thought to be essential for the effective delivery of the intervention, recruitment criteria specified that facilitators should be married women with at least one and a half years of experience in community work and a minimum of 12 years of schooling. To enhance acceptance in the community, facilitators had to live in the local area and be familiar with local culture and customs.

Facilitators received seven days of training in facilitation techniques, community mobilisation and participatory and communication skills. They were also trained in the use of picture cards and flip charts to convey simple messages and stimulate discussion. Based on field observations and facilitators' requests, refresher training was given six months after initial training. Frequent supervision by senior project staff provided on-going feedback and opportunities to review the status of facilitators' work and intervention delivery. Particular emphasis was given to facilitators to motivate and involve newly pregnant and reproductive-aged women in the women's group intervention.

Under the supervision of nine coordinators, the 45 facilitators catalysed community mobilisation, each running 18 women's groups in a month. They also liaised with community leaders, non-governmental organisations and other community based organisations and health care providers.

### Mapping

Using structured guidelines, local youth volunteers carried out village mapping. This included identification of existing women's groups and maternal and neonatal health projects run by other organisations in the intervention unions, health care providers, trained and un-trained traditional birth attendants and non-governmental organisations. Mapping also identified key local leaders for intervention initiatives. The facilitators conducted initial household visits to identify women of reproductive age and pregnant women, explained the intervention objectives to them and encouraged them to participate in women's group activities.

### Community orientation

Following mapping, informal meetings were held between project staff and community members to build rapport and to motivate local leaders to support the intervention. These meetings explained the intervention objectives, the role of facilitators and how the women's groups might work alongside local resources to improve maternal and neonatal health. The orientation meetings were also an opportunity to identify women interested in participation in the women's group meetings. A subsequent round of household visits was conducted to ensure that all women of reproductive age and pregnant women in the community were informed about the women's group intervention. Newly formed women's groups met for the first time in January 2009 and began a 30-month participatory action cycle on maternal and neonatal health.

### Group membership and participation

Women's group members are registered volunteers who should be ever-married women of reproductive age and are expected to directly participate in the women's group's activities and lead the implementation of strategies. There is a maximum of around 25 members per women's group. However, every women's group meeting is open to participation by non-members from the community, including men, who are interested in the meeting content but are unwilling or unable to volunteer as women's group members. These non-member participants are considered crucial to the community-wide acceptance, dissemination and broad participatory nature of the intervention.

### Targeted participation and dissemination

Participation among women of reproductive age and pregnant women in women's groups is a crucial factor for an effect on health and healthy practices, yet household responsibilities and cultural restrictions on women's ability to freely attend meetings are a barrier in many areas. As such, explicit strategies were employed to attract women of reproductive age, pregnant or newly married women to groups. These strategies included asking women at meetings to volunteer their pregnancy status and to identify anybody who they knew to be pregnant or newly married in the community. Traditional birth attendants, community health workers and community nutrition workers were also asked to inform facilitators if they were aware of women who might benefit from the intervention. Facilitators then visited these women in their homes and explained to them and influential family members (e.g. mothers-in-law, elders and husbands) the nature and objectives of the women's groups, encouraging them to participate. Women who remained unable or unwilling to participate were offered home visits by women's group members or facilitators to share key health messages and discussion topics raised during the meetings, which allowed diffusion of information.

Participation of young, newly married or pregnant women in the women's groups was difficult in some areas where traditional and conservative attitudes prevail or where there was resistance among influential community and religious leaders to the concept of women's groups. Several meetings between senior project staff and these community and religious leaders gradually broke down barriers, to some extent, with one particular success resulting in recommendations from a local Imam during religious services that people in the community should attend the groups.

### Measuring scale-up

An annual household listing is conducted in all study areas to provide information on the total population and age and sex distributions. In addition, a prospective community-based surveillance system of all births and maternal and neonatal deaths has been operational in study areas since 2004 [7,22]; all women who deliver in the study areas are interviewed about their pregnancy and delivery experiences, including any participation in women's groups. Simultaneously, a process evaluation system gathered information on the delivery and receipt of the women's group intervention. Structured forms were used in the process evaluation by facilitators to prospectively capture information on attendance at women's group meetings and the pregnancy status of all participants. In 2009, a one-off socio-economic and demographic survey was conducted with women's groups' members to gather data on their age and background characteristics. All household, surveillance and process evaluation data were subject to field quality control procedures before being entered, verified and stored in electronic databases.

Data from the community-based surveillance and process evaluation systems collected between January 2009 and June 2010 as well as data from the household-listing survey conducted in 2009 were used to calculate intervention coverage. Total population coverage was calculated by dividing the total population in the intervention areas from the household listing by the total number of women's groups. Similarly, the intervention's coverage of reproductive-aged women is calculated by dividing the number of women of reproductive age in the intervention areas from the household listing by the number of women's groups.

To calculate the proportion of women of reproductive age who are women's group members, the number of ever-married women's group members in reproductive age from the 2009 process evaluation socio-economic survey was divided by the number of ever-married women of reproductive age in the intervention areas from the household listing. The intervention's coverage of pregnant women was estimated using the community-surveillance data by dividing the number of deliveries to women that attend women groups by the number of deliveries in intervention areas.

Intervention dosage received at the population level was calculated by dividing the number of meetings actually attended by each group member by the total number of meetings implemented as part of the participatory action cycle intervention.

### Ethical approval

Scale-up and the evaluation of its impact was approved by the University College London Research Ethics Committee (ID Number: 1488/001) and by the Ethical Review Committee of the Diabetic Association of Bangladesh. Informed verbal consent from the interviewee was obtained before any data were collected.

## Results

Recruitment and training were considered successful and facilitators' skills in leading women's group meetings generally improved over time. There were some difficulties in meeting education and marital status criteria for recruitment due in part to household responsibilities, and the lack of availability of educated women, who were often engaged in better-paid jobs or positions offering greater benefits. Staff turnover was reasonably high, with 18 out of the 36 recruited facilitators resigning. Turnover mainly occurred in the first three months following recruitment and was primarily due to issues of family constraints on freedom of movement or offers of more competitive employment opportunities. This led to a revision of recruitment strategies, in that shortlisted candidates worked in the field on a voluntary basis for seven days before officially being appointed as facilitators.

**Figure 2:**
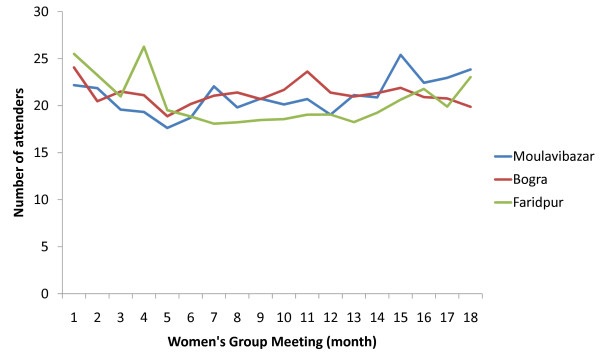
**Average attendance of participants (members and non-members) at women's group meetings by district in intervention areas**.

In a population of 243,341 people in the nine intervention unions, 648 new women's groups were added to the pre-scale-up 162 groups. All 810 groups continue to run to date. The population coverage is estimated at one women's group per 300 population (Table 1), an approximately five-fold increase in coverage relative to pre-scale-up levels. Following scale up, there is approximately one women's group per 57 ever-married women of reproductive age, compared to one group per 283 prior to scale-up and 23% of the 45,820 ever-married women in reproductive age living in the intervention areas are women's group members. Approximately 29% of women who gave birth and were interviewed as part of the community-surveillance system during the period January 2009 to June 2010 reported attending women's groups, compared to 3% prior to scale-up. Average attendance at each women's group meeting in the newly-formed groups is shown in Figure 2.

**Table 1:** Population coverage of the women's group intervention

Coverage Indicator		TOTAL
Number of Women's Groups	Pre-scale-up	162
	
	Newly formed	648
	
	Total	810

Population in 2009		243,341

Ever-married women in reproductive age in 2009		45,820

Population Coverage (Total population/number of women's groups) *(2009 pre-scale-up value)*		300 *(1502)*

Coverage of reproductive-aged women (Total population of ever-married women in reproductive age/number of women's groups) *(2009 pre-scale-up value)*		57 *(283)*

% of reproductive aged-women who are women's group members *(2009 pre-scale-up value)*		23 *(9)*

% deliveries to women attending women's groups *(2009 pre-scale-up value)*		29 *(3)*

Intensity of the exposure to the intervention among women's group members in 2009 was estimated at 70%, meaning that, on average, women's group members were exposed to 70% of the intended dosage for 2009, i.e. an average of 8 out of 12 meetings were attended.

## Discussion

For both women's groups and community health worker interventions, coverage is thought to be a critical factor for a positive impact on neonatal mortality rates [14]. Simple quantitative data indicate considerable success in the scale-up of the women's groups in rural Bangladesh. We showed an approximately five-fold increase in population coverage, a 2.5-fold increase in the women's group membership among women of reproductive age and a 10-fold increase in the proportion of women who gave birth and reported attending a women's group. Scale-up was achieved without financial incentives for women and without any increase in managerial staff. Nevertheless, enhancement of already strong operational capabilities and institutional knowledge within our Perinatal Care Project (PCP) was crucial for the success of scale-up. Furthermore, PCP works under the auspices of BADAS, whose positive reputation as the largest non-governmental healthcare provider in Bangladesh facilitated acceptance of the intervention.

The specific impact of the scale-up on mortality rates is yet to be determined through an ongoing cluster-randomised trial [17], which includes detailed analysis of process indicators of delivery and receipt of the intervention. Nevertheless, documenting the process and success of scale-up is itself important if development initiatives are to foster participation and remain accountable to communities, and to avoid cumbersome organisational structures that are detached from their grassroots bases [23]. This paper does not presume to provide the ultimate solution to such challenges, but rather shares experiences of a single intervention in a resource-poor setting. Though limited to just nine unions in rural Bangladesh, these experiences and the described principles and processes of scale-up and its measurement are likely to be relevant to the wider development community, notwithstanding the need for intervention- and context-specific alterations and for programme flexibility.

Experience of delivery of the intervention on a smaller scale enabled PCP to meet the majority of requirements for successful scale-up as described by Simmons et al. (2006)[24] and summarised by Gilson and Schneider (2010)[19]. For example, clear messages about the objectives and advantages of women's groups were communicated to the community, while mapping exercises, household visits and community orientation meetings ensured early involvement through personal contact. Similarly, recognition of the importance of locally recruited and trained facilitators to manage groups, the participatory nature of the intervention, and active diffusion of key messages through household visits and community, governmental and non-governmental networks were central to the scale-up process. Concurrent systematic monitoring of the process and outcomes of scaling up further met Simmons et al's (2006)[24] and Gilson and Schneider's (2010)[19] recommendations to use evidence to guide and evaluate scale-up.

The scaling-up process described is a logical sequence and represents more or less the order in which various stages were initiated. In practice, however, the linear presentation is artificial and it is important to recognise that scaling-up is a dynamic, non-linear, iterative process whereby the various phases may occur simultaneously, in different orders, have feedback loops and may have to be repeated or revisited [18]. Feedback systems that allow for periodic programme corrections and continued innovation are central to successful scale-up and require managerial flexibility and strategic flair [19,25].

Training and capacity building of staff is crucial for a thorough understanding of the group process and a sense of ownership at the grassroots level. Recruitment and selection of appropriate staff is critical, as are planned strategies to cope with staff turnover. Local recruitment was vital in maintaining effective relationships with the local community through familiarity with local customs, knowledge and beliefs. Recruitment criteria, however, cannot be rigid in areas of low literacy, low education and where there are gender barriers to employment. The resource-poor, rural context of our study area and an initial lack of appreciation by project managers for the need for family understanding of facilitators' roles increased vulnerability to staff turnover at the beginning of scale-up. This necessitated greater flexibility in recruitment criteria and processes, including a probationary period of field exposure, which ultimately minimised disruption caused by staff turnover. Effective systems of supervision, review and refresher training of project staff are likely to enhance delivery and sustainability of any intervention and therefore the success of any scale-up initiatives.

Various definitions of scaling-up exist, relating to complexity, impact and interactions with other organisations, but the concept of scaling-up as expansion of coverage is the most common [15,23]. Geographical expansion is associated with particular challenges such as increasing distance from project headquarters, and larger areas and organisational structures to manage. Notwithstanding the introduction of groups into new villages in the existing unions, we did not expand geographically. Rather, our experience was an increase in the intensity of intervention in the same areas to increase population coverage. Some of the issues around effective geographical expansion and increased intensification are the same, however. In each case, scale-up does not mean exact duplication of pre-existing strategies. In our scale-up, lessons were learned from the previous, smaller-scale implementation of women's groups but existing structures and procedures were not always copied. For example, the schedule of group meetings and content were revised before scale-up to emphasise participation of women of reproductive age, and especially pregnant women. As a flexible replication of similar women's groups interventions in Nepal and India we endorse the recommendation that replication as a path for scaling up is only likely to work if done flexibly [23].

Strategies to measure the success of scale-up depend on specific objectives and anticipated outcomes. In relation to population coverage or intervention expansion, specific indicators of success, and how they are measured, are essential. There is no one-size-fits-all strategy for monitoring and evaluation and different methods have differing degrees of complexity and resource demands [26,27]. Data from three different sources were used to measure the success of our scale-up, a particular strength of this initiative.

The capacity needed for such monitoring and evaluation systems should not be underestimated, however. Complex systems add to the cost of interventions considerably and are vulnerable to the limitations of population survey methods, such as recall and reporting biases [18,27]. Furthermore, direct measurements of certain phenomena are not always straightforward or possible. For example, intervention coverage among pregnant women in the women's group intervention could not be measured directly as it was not possible to accurately measure the pregnancy status of all reproductive-aged women. The proportion of births that occur to women who report attendance at a women's group was therefore used as a proxy measure of coverage among pregnant women. This estimation depends on accurate reporting of women's group attendance and on all pregnancies ending in delivery, which is unlikely to be true. Nevertheless, provided that limitations are acknowledged and measurement methods are consistent pre- and post-scale-up, utilising proxy measures and actual data are more informative than entirely modelled estimates. Alternative methods for estimating coverage of an intervention among pregnant women with varying degrees of complexity, cost and data demands are described and discussed elsewhere [28].

Although the sustainability of women's groups is yet to be formally evaluated, unpublished data suggest that a high proportion of groups in Nepal and Malawi are running long after withdrawal of project financial support. Successful delivery of the intervention as planned during the first 18 women's group meetings may be considered indicative of institutional sustainability in terms of ongoing capacity to lead training, maintain the infrastructure, equipment and supplies and provide the necessary inputs and environment for implementation. Similarly, fairly consistent attendance levels at the women's groups (Figure 2) and reasonable population exposure estimates indicate programmatic sustainability from a community participatory perspective. Continued capacity building of women's group members, perhaps with a focus on facilitation methods for example, may obviate the need for paid facilitators and thus enhance the long-term financial sustainability of the intervention, whilst political sustainability can only be achieved through enhanced communication and advocacy efforts at local, national and global levels.

The ability to influence national policy from local initiatives is therefore a final determinant and indicator of successful scale-up [23,25]. Before the women's groups became active, considerable time was spent identifying available services and other governmental and non-governmental organisations in the selected unions to communicate and build understanding and trust within the local community. These efforts enhance the participatory nature of scale-up as opposed to being purely driven by the implementing institutions or experts. In the women's groups, establishing links with other key players was beneficial in a number of ways, not least by involving traditional birth attendants and community health workers in raising awareness of the intervention among pregnant women encountered during their routine activities. We cannot objectively assess the success of wider stakeholder engagement in terms of bridging gaps and influencing policies, but these efforts are undoubtedly important for the acceptability and long-term sustainability of scale-up.

## Conclusion

Strong operational capabilities, clarity of scale-up and intervention objectives and wide stakeholder engagement were critical to the successful scaling-up of the women's group intervention in rural Bangladesh. It was possible to increase community engagement with the intervention without financial incentives and without an increase in managerial staff. Monitoring and feedback systems that allowed periodic programme corrections and continued innovation were central to successful scale-up and required programmatic and operational flexibility.

## List of Abbreviations

BADAS: Diabetic Association of Bangladesh; PCP: Perinatal Care Project; MDG: Millennium Development Goal.

## Competing interests

The authors declare that they have no competing interests.

## Authors' contributions

All authors contributed to drafting and revising the manuscript. In addition: TN - implementation of women's groups, substantial contribution to the overall scale-up process; KA - overall management of the PCP project, including the scale-up process, process evaluation and monitoring processes; BHA - design, management and interpretation of process evaluation data systems; LY - scale-up and process evaluation processes and interpretation of data; SS - capture, management and reporting of monitoring and evaluating data and data processes; AK, AP, TH and AC - technical support to scale-up activities and critical oversight to the documentation, interpretation and reporting of scale up and monitoring processes; EF oversaw the conceptual development of the manuscript, interpretation and presentation of results and the overall development and revision of the manuscript. All authors have read and approved the final manuscript.

## Endnotes

^i ^The definition of ever-married used in this paper includes married or divorced but not widowed women.

^ii ^Community nutrition workers have a minimum of 8 years of basic schooling and have received 21 days training on nutrition and counselling.

^iii ^A Good Practice Guide [21] to delivering the women's group intervention, which may facilitate further replication and scale-up, is available at: http://www.wcf-uk.org/knowledge/wcf-publications/45-wcf-publications/363-good-practice-guide.

## Pre-publication history

The pre-publication history for this paper can be accessed here:

http://www.biomedcentral.com/1471-2393/12/5/prepub
